# Editorial: Advances in Osteoimmunology

**DOI:** 10.3389/fimmu.2019.02595

**Published:** 2019-11-13

**Authors:** Claudine Blin-Wakkach, Teun J. de Vries

**Affiliations:** ^1^Université Côte d'Azur, Nice, France; ^2^CNRS UMR7370, Laboratoire de PhysioMédecine Moléculaire, Nice, France; ^3^Academic Centre for Dentistry Amsterdam, University of Amsterdam and VU University, Amsterdam, Netherlands

**Keywords:** osteoimmunology, inflammation, osteoclast, T cell, bone marrow

The association between chronic inflammation and bone destruction has long been recognized, but the molecular bases of the underlying mechanisms were identified only 20 years ago with the discovery of the essential role of the RANK/RANKL axis in bone and immune cell physiopathology [reviewed in (1)]. From this moment, the term “osteoimmunology” was proposed to define a new discipline covering the interplay between the bone and the immune system (2). Osteoimmunology has become an essential discipline for the study of a huge variety of inflammatory diseases such as rheumatic diseases, aging as manifested in osteoporosis, chronic inflammation such as inflammatory bowel disease, bone infection and bone healing such as is apparent in periodontitis and after surgery, as well as for cancer. Publications related to osteoimmunology are steadily increasing in number and cover fields as varied as immunology, endocrinology and metabolism, cell biology, biochemistry, rheumatology, experimental medicine, pharmacology, dentistry, biomaterials, and hematology (from Web of Science). This Research Topic brings together 24 contributions by 162 authors from all over the world, from North- (10) and South-America (21), Europe (113), Asia (3), and Australia (15). When summarizing all contributions, the topic has deepened our understanding on four topics in particular.

## Components of the Immune System Controlling Osteoclast or Osteoblast Differentiation and Function

The first major question in osteoimmunology has been to understand how the immune system controls the differentiation and activity of bone cells. Initially, an important role was attributed to Th17 cells that produce RANKL, IL-17, and TNF-α all increasing osteoclast formation, as reviewed in this topic in the context of arthritis (Coury et al.), inflammatory bowel disease (Madel et al.) and periodontal diseases (Alvarez et al.). Biphotonic microscopy became an important tool that enables visualization of the dynamic interaction between osteoclasts and T cells, as presented by Hasegawa et al.. The B cell lineage also plays an important role in controlling osteoclastogenesis. As reviewed by Coury et al., autoantibodies against citrullinated proteins (ACPA) mediate bone destruction in rheumatoid arthritis. The underlying mechanisms linking ACPA and osteoclastogenesis in arthritis were further explored in the review of Steffen et al.. The role of the adaptive immune system appears therefore essential in osteoimmunology. This was further emphasized in two papers from the group of Schmidt-Bleek. Bucher et al. demonstrated that, during aging in mice, the acquisition of a more experienced adaptive immune system alters the bone structure and mechanical properties and decreases the bone healing capacity of the mice. The same group (Wendler et al.) reported that the immune suppressive drug Iloprost stimulates the osteogenic capacity of mesenchymal cells and bone healing by reducing the production of proinflammatory cytokines by CD8^+^ T cells and modulating the M1/M2 balance in macrophages.

Nowadays, the effect of the immune system on bone cells appears much more complex, and beside T cells, many other immune cells also influence bone formation and/or resorption. Of course, myeloid cells greatly contribute to osteoimmune interactions mainly because some of them represent osteoclast progenitors. In a systematic literature review, de Vries et al. highlighted two common cell types participating in osteoclastogenesis in chronic diseases and bone metastasis: blood CD16^+^ monocytes as major osteoclast progenitors, and T cells producing TNF-α that support pathological osteoclastogenesis. The origin of osteoclasts from myeloid cells was further reviewed by Madel et al.. They pointed out that dendritic cells contribute to osteoclast formation in pathological conditions related to chronic inflammation and cancer, always in the presence of high levels of IL-17, TNF-α, and RANK-L.

Two reviews present the importance of macrophages in osteoimmunology. Humbert et al. reassessed the reciprocal interactions between macrophages and mesenchymal stromal cells that modulate immune suppression and bone regeneration in bone healing after calcium-phosphate implant transplantation. Biguetti et al. demonstrated using Ti-implants that Damage Associated Molecular Patterns (DAMP) such as HMGB1 and Rage are essential for osteointegration by controlling the balance between M1 and M2 macrophages. As discussed by Pieters et al., macrophages produce extracellular vesicles (EVs) that mediate their interaction with bone cells. Among the various compounds carried by these EVs, alarmins, which are DAMPs released upon stress or inflammation, influence bone remodeling, decreasing or increasing bone resorption and formation depending on the content of the vesicles. EVs also carry miRNAs that are able to control bone cell differentiation. The role of miRNAs in osteoclastogenesis was further considered in a review by Lozano et al..

These data emphasize the importance of danger signals in osteoimmune interactions. This was further discussed by Souza and Lerner who, showed that Toll like receptors that recognize signals from bacteria and other microorganisms participate in the control of osteoclast, osteoblast, or MSC differentiation and function. In their review, Seebach and Kubatzky showed that implant-associated bone infection induces an immune-compromised environment where bacteria can persist, resulting in increased bone resorption. In this environment, different immune cells—including osteoclasts—may participate in an immune-suppressive environment that favors the chronicity of infection.

## Control of Inflammation and Immune Cells by Bone Cells

The interaction between the bone and immune system is reciprocal. Mesenchymal stromal cells have an important immunosuppressive function that participates in regulating inflammatory responses (Xiao et al.) and in bone healing (Humbert et al.). In rheumatoid arthritis, Luque-Campos et al. analyzed the capacity of MSCs to restore the balance between inflammation and tolerance, which is of high interest for therapeutic purpose. Moreover, cells from the mesenchymal lineage are a major component of hematopoietic niches. Using lipodystrophic mouse models, Wilson et al. demonstrated that adipocytes are required for maintaining an environment that favors the retention of hematopoietic progenitors in the bone marrow.

An emerging field in osteoimmunology is the immune function of osteoclasts. Madel et al. provided the first review on this novel aspect of osteoclast activity. In line with the different origins of osteoclasts, they discussed the heterogeneity of mature osteoclasts as well as their function as innate immune cells. They showed that besides their bone resorption activity, osteoclasts are immuno-competent cells able to initiate T cell responses toward tolerance or inflammation depending on their context and origin (3). This opens new research avenues on the heterogeneity of osteoclasts in steady state and in chronic inflammatory conditions.

## Signaling and Regulatory Pathways in Osteoimmunology

At the molecular level, the topic has contributed in refining osteoclast signaling pathways in the context of the immune system and diseases with bone destruction. Sobacchi et al. updated us on the importance of RANK-RANKL signaling not only for osteoclast formation in bone (4), but also for T-cell maturation in the thymus. Using various TNF-α and RANKL knock-out, and overexpression mouse models, Papadaki et al. demonstrated that overall, arthritis was weakened in the absence of RANKL, but increased osteoclast formation at the pannus area was observed when TNF-α was overexpressed even in the absence of RANKL, confirming a RANKL-independent osteoclast formation (5). In contrast, overexpression of TNF-α was not able to compensate osteopetrosis in the absence of RANKL, indicating that disease-associated osteoclasts and turnover or physiological osteoclasts may have a different dependency on RANKL or TNF-α for their formation (as discussed in de Vries et al.; Madel et al.).

Cytokine signaling toward osteoblasts and osteoclasts has always been a key topic in osteoimmunology (6). Persson et al. interfered with family members of the gp130 receptor cytokine family in osteoblasts. When activating Shc1, Oncostatin M-mediated RANKL upregulation and subsequent osteoclastogenesis through interference with STAT3 signaling was achieved. Various studies suggest a role for inflammation in the onset of formation of heterotopic bone (7). In a model for spinal cord injury-induced heterotopic ossification, Alexander et al. showed that injured muscles display increased STAT3 signaling, activating JAK1/2 tyrosine kinases. When inhibiting this pathway, heterotopic ossification was diminished.

Two review articles described the importance of S1P-S1PR signaling in egression of immune cells to inflammatory bone (Hasegawa et al.; Xiao et al.). One of the future challenges in the osteoimmunology field is to map the osteoclast-immune cell-interactions. When do and what kind of T cells interact with bone resorbing osteoclasts, and will these stimulate or inhibit their activity? The life cell imaging of bone-immune cell interactions (Hasegawa et al.) as developed by the group of Ishii (8, 9) will certainly assist herein. Syk is a non-receptor tyrosine kinase critically involved in signaling by various immune receptors. Mouse models where hematopoietic lineage or osteoclast specific knock-out of Syk is accomplished, develop osteopetrosis, demonstrating the role of Syk in osteoclasts (Csete et al.).

## Pathological Implications of Osteoimmunology

For the understanding of the pathophysiology of inflammatory bone diseases, our series of articles has contributed in highlighting the role of osteoimmunology in various diseases. First of all, possible common ground for the various inflammatory bone diseases in peripheral blood was found at the level of monocyte precursor type priming within the circulation by inflammatory cytokines such as TNF-α and a role for activated RANKL and/or TNF-α expressing T cells (de Vries et al.). Secondly, common osteoimmunology ground was searched for in diseases of the oral cavity such as periodontitis, oral cancer and degradation of the temporomandibular joint (Alvarez et al.).

For rheumatoid arthritis, our series had three review contributions, one general review (Coury et al.), and two more specialized ones describing a putative role for mesenchymal stem cells (Luque-Campos et al.) or autoantibodies (Steffen et al.) in disease modulation.

Obesity is a growing health care concern in Western society, a condition associated with an altered immune system (10, 11). Obese children display a deviant monocyte subpopulation distribution and concomitant increased osteoclast formation, which can be modulated with dietary substances such as sweet cherry polyphenols that reduce RANK-L and TNF-α production (Corbo et al.). At the other side of the spectrum, in two mouse models that lack adipocytes, hematopoiesis moved outside the bone marrow to the spleen and liver (Wilson et al.).

Bone infections, such as around implants or around teeth, may alter the immune system-driven osteoclast formation. Osteoclasts ultimately may contribute to implant or tooth loosening when not treated properly. Seebach and Kubatzky have investigated whether immune modulation could be a therapeutic target for chronic bone infections. Osteoclast precursors such as monocytes originate from the bone marrow or blood. Once at the site of a bacterial infection, they make a differentiation decision, either into macrophages, combating the infection, or into osteoclasts. These monocyte or osteoclast precursor cells respond with toll-like receptors to bacterial products. This toll on the route when egressing from the circulation determines the fate and can be both inhibitory and stimulatory (Souza and Lerner). Despite all attempts of cell biologists to mimic inflammation in a Petri dish, the influence of mechanical loading is often neglected. Fahy et al. have taken up the challenge to map the contribution of mechanical loading and found that mechanically loaded monocytes secrete a different repertoire of cytokines than the unloaded ones.

## Concluding Remarks

Bone quality and bone healing are age-dependent, with a decreased osteogenesis and an increased osteoclastogenesis over time. Parallel to this, the immune system also changes over time and can be “learned” or “naïve.” In order to dissect both components, bone strength and *in vitro* osteogenic capacity were analyzed in mice of various age, and the effect of learned and naïve immune system was analyzed. Supernatants of immune cells inhibited osteogenic capacity of mesenchymal stem cells, stronger so in older mice and in immune-stimulated mice (Bucher et al.). Suppression of inflammatory milieu at early stages of bone fracture may improve bone repair (Wendler et al.). Inflammatory processes also take place during early phases of implant osseointegration. Biguetti et al. have assessed the role of HGMB1 and RAGE in titanium osseointegration and demonstrated that activity of these immune modulators is essential for successful osseointegration. Many devices used for implantation are coated with calcium-phosphate. Humbert et al. reviewed the state-of-the-art of these implants in conjunction with co-transplantation of mesenchymal stem cells, which may provoke positive immune modulation.

**Figure 1:**
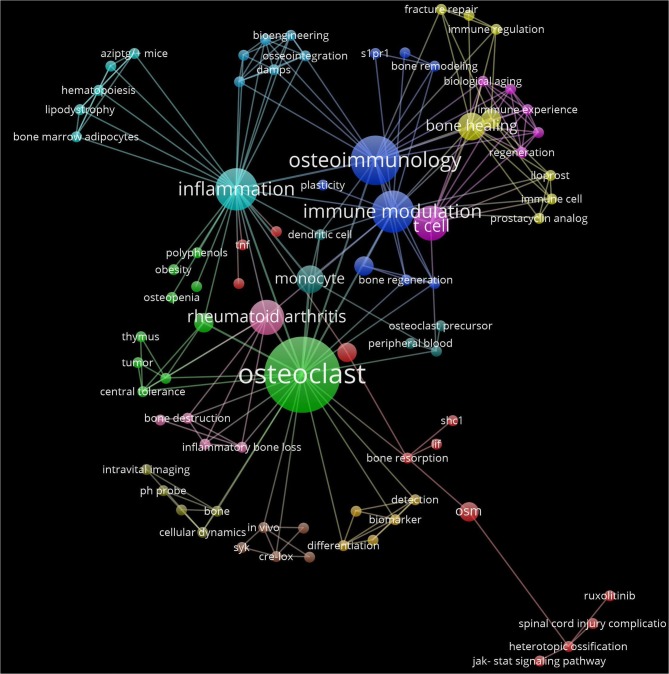
Interaction network of articles from the Research Topic “Advances in Osteoimmuology”. The network is built on the keywords from the 24 articles using VosViewer (http://www.vosviewer.com/) (ref https://doi.org/10.1007/s11192-009-0146-3). Keyword colors are determined by the cluster to which they belong. Each line represents an interaction among the keywords Distance between keywords approximately indicates their relatedness in network. The size of each keyword label and circle depends on the weight of the keyword.

For 20 years, osteoimmunology has more and more found its way into the field of immunology, even at the undergraduate level (12). The topic “Advances in Osteoimmunology” shows great diversity in the themes that were addressed. Relatively new is the attention for implants and the role of immune cells and bone cells. The key cell still seems to be the osteoclast (Figure 1). Concerning a deeper understanding in the pathophysiology of osteoclasts formed under the control of the immune system, specific markers, of for instance, osteoclast membrane markers such as CX3CR1 [Madel et al.; (3)] or blood-derived precursors such as miRNAs (Lozano et al.) could generate disease-specific fingerprints. However, one can never be certain about the fate of these latter circulating markers. Generation of osteoclasts from monocytes from patients will only partially provide fingerprint answers, since only very few cells turn into multinucleated osteoclasts in any *in vitro* experiment. Therefore, isolation and characterization of pure osteoclasts, such as which has recently been described (13) isolated from bone biopsies, may further advance the field. High-throughput technologies such as single cell RNAseq analysis (14) are bound to be successful in future research, deciphering the phenotypic and functional diversity of bone marrow cells (15) including for osteoclasts. This will pave the road for understanding of deregulated osteoimmune interactions and more specific targeting of cells participating in pathological bone loss.

## Author Contributions

CB-W and TV designed the project and wrote the manuscript.

### Conflict of Interest

The authors declare that the research was conducted in the absence of any commercial or financial relationships that could be construed as a potential conflict of interest.
